# Does gender diversity mediate the relationships of diversity beliefs and workplace happiness?

**DOI:** 10.3389/fsoc.2024.1384790

**Published:** 2024-08-01

**Authors:** Shafiqul Islam, Md. Jahangir Alam, Maileenita Penalba

**Affiliations:** ^1^School of Business and Economics, United International University, Dhaka, Bangladesh; ^2^Department of Management, Jatiya Kabi Kazi Nazrul Islam University, Mymensingh, Bangladesh; ^3^Department of Economics and Political Science, University of the Philippines Baguio, Baguio, Philippines

**Keywords:** diversity beliefs, gender diversity, work engagement, organizational commitment, workplace happiness

## Abstract

Though its intensity varies across cultures, practicing diversity beliefs has become increasingly prevalent in contemporary business organizations. Traditionally, diversity encompasses various aspects such as gender, education, religion, language, age, ethnicity, culture and personality orientation. The current study has demonstrated to identify the mediating effects of gender diversity on diversity beliefs and workplace happiness. The targeted population is made up of full-time Bangladeshi employees working in both public and private organizations. The researchers distributed a questionnaire to 500 employees and obtained 320 valid responses, resulting in a response rate of 64%. The study used the Structural Equation Modelling (SEM) technique to assess the mediating effects and direct effects among the variables. The results demonstrate that gender diversity significantly mediate the associations between diversity beliefs and workplace happiness. This means that employees are more likely to be happy with their jobs if the workplace reflects gender variety in its workforce. The study further demonstrates that workplace happiness significantly affects employees’ job satisfaction, engagement and commitment. The present research foregrounds that firms and concerned authorities must increase their various attempts to establish gender-equal policies that appear to be more effective for diversity practice and workplace happiness in work organizations.

## 1 Introduction

The idea of diversity has become more widely accepted in the management literature over the past 20 years. While some organizations view it as a boon to creativity and competitive advantages, others view it as a barrier, bias, and limitation (Turi et al., 2022). Traditionally, diversity encompassed various aspects such as gender, education, religion, language, age, ethnicity, culture, and personality orientation (Kundu, 2003). Currently, various employers have viewed it as a tool for expanding into new markets and a motivation for their employees’ continued paid work (Harrison and Sin, 2006). Additionally, diversity is necessary for organizations to make rational decisions and foster a welcoming atmosphere where employees’ ideas are respected, consequently encouraging them to consider the advantages of their work (Roberson, 2019; Amin et al., 2022). Therefore, employees have been increasingly joining organizations that embrace diversity and respect differences. In order to create an inclusive work environment that promotes justice, tolerance, and equal chances for employees regardless of their differences such as gender identity, religion or age, organizations have started assessing and upgrading their diversity-related policies and actions on a regular basis (Blouch and Azeem, 2019; Turi et al., 2022).

This study draws from various literature on diversity and how diversity is enacted and managed in the workplace. Diversity refers to the physical and sociological differences among individuals within a workforce (Roberson, 2019). As diversity becomes increasingly seen as an ethical mission or goal of any organization, many studies have focused on understanding its impacts on employee productivity and overall organizational performance (Saxena, 2014; Mateescu, 2017; Ayega and Muathe, 2018; Cletus et al., 2018; Gomez and Bernet, 2019; Dale-Olsen and Finseraas, 2020). Highlighting trends and challenges in implementing diversity management is also common in diversity research. For example, many studies have argued for the involvement and intervention of top leadership to ensure the success of diversity initiatives and policies (McCann et al., 2017; Croitoru et al., 2022). Scholars have also identified common barriers to effective diversity management. These include discontinuity between diversity policy and practice (Allison, 1999) and structural, cultural, and communication issues within the workplace (Rangarajan and Black, 2007; Wilson et al., 2016; Aragon and Kim, 2021; Lang, 2021). Furthermore, there are lingering questions about how diversity can be successfully managed in the face of increasing multiculturalism and identity politics, which organizations must acknowledge and consider in their strategic planning. This study, therefore, asks, does gender diversity mediate the relationships of diversity beliefs and workplace happiness? By looking at these variables within the Bangladeshi context, this study fills the gap in gender diversity research that tends to focus more heavily on measuring organizational performance and equalizing compensation, recruitment, and leadership roles among genders and less on investigating employee happiness and well-being (see Belingheri et al., 2021).

The study proceeds by surveying full-time Bangladeshi employees working in both the public and private sectors to learn more about their experiences with diversity management and how gender diversity affects their views of these policies and practices, as well as their overall satisfaction with their workplace. This is because diversity management is new to Bangladeshi organizations, and diversity research is still in its infancy (Uddin and Chowdhury, 2015; Khan et al., 2023). Previous studies have shown that corporate bias arises from a lack of diversity training and awareness of different points of view, especially in developing countries with strong social and cultural ties like Bangladesh (Rahman et al., 2012; Uddin and Chowdhury, 2015; Rezina and Mahmood, 2016). According to Uddin and Chowdhury (2015), diversity in the workplace entails having individuals from different cultural and socioeconomic backgrounds working together. Effective diversity management ensures that all members of the diverse workforce are able to contribute to the fullest extent of their abilities in a setting free from bias and discrimination. Bangladesh is a predominantly male-dominated nation where there is a lack of diversity in organizational culture, particularly when it comes to gender equality in the workplace (Rezina and Mahmood, 2016; Rahman et al., 2018). Nonetheless, public and corporate policies have been gradually shifting in light of the fact that women constitute half of the population in the country and thus amount to half of the workforce. Furthermore, women’s tertiary education enrolment in Bangladesh has continued to rise over time, demonstrating their growing share of the human capital (Hossain, 2016; Uddin and Xie, 2019). Therefore, it is opportune to investigate if gender diversity is a successful practice in Bangladeshi firms due to market demand and if it mediates the workers’ happiness net of diversity management in the workplace.

## 2 Literature reviews and hypothesis

Diversity management in the workplace can be explored using the social exchange theory. In studying organizational behavior, the social exchange theory proves useful in determining mutual or reciprocal arrangements that may build trust and loyalty between organisation and employees (see Cropanzano and Mitchell, 2005). The theory further posits that “employees reciprocate in terms of their perception and performance with respect to treatment and information received from their organization” (Liaquat and Mehmood, 2017). Other studies have also used this theory to explore how recognizing and valuing individual differences can increase overall performance and satisfaction within organizations pointing to the crucial role of leadership in diversity practice (Gould-Williams and Davies, 2005; Ali and French, 2019; Nachmias et al., 2022). Further, the attitudinal theory can also be employed to explain employees’ level of satisfaction with and commitment to the organization. This theory allows us to investigate the cognitive, behavioral, and affective components of organizational dynamics (Ajzen and Fishbein, 1973). The study draws from these theories in analyzing survey results later in the paper.

Meanwhile, a vast number of literature argues that promoting diversity in the workplace is often linked to business competitiveness and efficiency. As per the tenets of management philosophy, the purpose of diversity management is to optimize organizational performance and enhance the overall work experience by capitalizing on a range of abilities, beliefs, and perspectives. Diversity, however, is a broad term that signifies a wide array of tangible and intangible differences that characterize modern populations. While diversity can be regarded as an asset, “[i]f not managed properly, it [also] has the potential to harm morale, intensify turnover and result in substantial communication problems” (Turi et al., 2022, p. 1). Thus, incorporating diversity can become a challenge to most organizations. In light of this, diversity management ought to be planned and methodical, involving the development and execution of policies and processes that foster an inclusive work environment (Gross-Gołacka et al., 2022).

### 2.1 Diversity management

Diversity management comes in various forms. It can manifest as gender quotas or other forms of affirmative action, diversity training and education, and anti-discrimination policies. There are also different ways to justify and promote diversity management. In their work on cultural diversity, for instance, Ely and Thomas (2001) offer three perspectives on managing workforce diversity. These are the integration-and-learning perspective, the access-and-legitimacy perspective, and the discrimination-and-fairness perspective. Each perspective offers a framework for understanding why and how cultural diversity impacts work experience. First, the integration-and-learning perspective posits that cultural diversity is a resource that organizations can tap into by intentionally making their workforce multicultural. The idea is that having a diverse workforce allows staff members to educate and learn from each other, facilitating individual and organizational change beneficial to the institution. Second, the access-and-legitimacy perspective embraces diversity, reflecting the multicultural nature of markets and constituencies. The aim is to gain access to and legitimacy from these diverse markets and constituent groups through their meaningful representation within the organizational hierarchy. Third, the discrimination-and-fairness perspective is “characterized by a belief in a culturally diverse workforce as a moral imperative to ensure justice and the fair treatment of all members of society” (Ely and Thomas, 2001, p. 245). This perspective is regarded as the basis of affirmative action programs that aim to guarantee recruitment and retention of minority groups.

These three perspectives illuminate why diversity management plays a significant role in organizational performance and development. This paper, however, focuses on gender diversity as one aspect of diversity management that needs further analysis within the Bangladeshi context. The following discussions highlight the key arguments explored in this study.

### 2.2 Diversity beliefs and gender diversity in organizations

Gender is one of the individual attributes considered when acknowledging and managing diversity. Organizations that value and promote diversity may focus on ensuring fair, if not equal, gender representation within their management structures. Although gender diversity may typically be understood as a representation of both men and women, especially in management positions, it can also signify meaningful inclusion of other commonly recognized and expressed gender identities (i.e., LGBT). However, our study focuses only on man and woman. Incorporating gender diversity in organizational management is also increasingly seen through its moral and business sense (see Campbell and Mínguez-Vera, 2008; Boulouta, 2013). Various studies, in fact, show that gender diversity within organizations can have positive effects on improving business performance, quality of decision-making, fraud mitigation, conflict management, and even investor confidence (Amin et al., 2022; see also Diaz-Garcia et al., 2013; Noland et al., 2016; Reddy and Jadhav, 2019; Hossain et al., 2020). Hossain et al. (2020), for instance, show that workplace diversity policies, specifically those that prohibit discrimination based on sexual orientation and gender identity, have positive effects on boosting innovation and performance among firms in the United States (US). Their study offers quantitative evidence suggesting that firms that enact diversity policies perform better than those without such policies. Diaz-Garcia et al.’s (2013) study also confirms that gender diversity is positively related to radical innovation in the organisation.

Furthermore, Elvi and Tunjungsari’s (2022) study of 100 employees from Indonesia’s telecommunication companies suggests that “[l]eaders with female gender will be more innovative and proactive than male in the same position” (p. 353). The study by Amin et al. (2022) also indicates that encouraged by the diversity beliefs, gender-diverse boards, meaning those that have a “female presence,” increase business confidence “due to strong monitoring skills, better decision making and effective leadership” (p. 28). Other studies confirm that gender diversity in the boardroom positively affects organizational performance for organizations that promote diversity beliefs (Kim and Starks, 2016; Lu and Herremans, 2019; Brahma et al., 2021). Thus, the central premise of this section is that gender diversity mediates diversity beliefs and organizational happiness. Although Western nations have encouraged diversity beliefs in promoting gender equality and equity to some extent, the practice of diversity beliefs and, hence, the promotion of gender diversity is still under dire scrutiny in Bangladesh. Considering this literature, the following hypothesis was generated to examine the Bangladeshi context.

H1: Diversity beliefs of the employees positively promote the gender diversity in the workplace.

### 2.3 Diversity beliefs and workplace happiness

In their qualitative study on workplace diversity in Bangladesh, Cho and Sultana (2017) present how diversity beliefs can facilitate workplace happiness. Based on a case analysis of the Bangladesh Rural Advancement Committee (BRAC), their findings reveal that deliberate inclusion of employees with different religious and cultural beliefs helps build a friendly environment within the workplace, which then “positively impacts [the employees’] physical and mental health and overall wellbeing” (Cho and Sultana, 2017, p. 11). Foma (2014) similarly argues that having cultural diversity in the workplace facilitates a healthy exchange of ideas between employees and eliminates stereotyping. Lambert (2016) further posits that “a climate that is not supportive of diversity may create a culture perceived by employees to lack organizational support, cohesion, and identity resulting in interpersonal communication breakdowns which can hinder the elements needed for innovation to exist” (p. 17). Lack of diversity beliefs in the workplace can, therefore, lead to both interpersonal and organizational problems that can significantly affect not just productivity but camaraderie and happiness among employees as well. Given these studies, this paper explores how diverse beliefs within Bangladeshi organizational settings affect workplace happiness.

H2: Diversity beliefs of the employees positively affect their workplace happiness.

### 2.4 Gender diversity and workplace happiness

While most studies on gender diversity focus on how it is linked to productivity, innovation, and effective leadership, gender diversity is also believed to affect workplace happiness. Mousa (2021), for example, proves that gender diversity is one of the predictors of workplace happiness. Analyzing survey data from three public universities in Egypt, Mousa’s (2021) study indicates that “female academics perceive, appreciate and respect diversity policies at their universities better than their male colleagues” (p. 9). This significant difference between male and female perceptions of diversity policies supports the hypothesis that gender diversity can, in fact, predict workplace happiness based on the level of active engagement, job satisfaction, and organizational commitment among employees. Dibobo et al. (2022) noted that the relative lack of gender and racial diversity in the automotive industry in South Africa contributes to feelings of discomfort among employees who report cases of unequal treatment, lower pay, and lack of opportunities to advance within their workplace. As a result, this lack of diversity within the organization leads to unhappiness and discontent, especially among rank-and-file employees. In their business case study, Azmat and Boring (2020) also suggest improving gender diversity policies to promote a fairer, safer, and more family-friendly working environment for women. Azmat and Boring’s (2020) evaluation of gender diversity policies specifically draws attention to how a lack of gender diversity may further entrench a corporate culture that breeds discrimination, sexual harassment, and the gender pay gap targeting women. Arguably, such a firm culture deeply diminishes happiness in the workplace. Conversely, a work culture that cultivates gender diversity can help create an environment that values women, recognizes their contribution, treats them fairly, and protects their rights and well-being, leading to workplace happiness.

H3: Gender diversity positively affects the workplace happiness of the employees.

### 2.5 Diversity beliefs, gender diversity and workplace happiness

Managing diversity in the workplace necessitates the inclusion of individuals and groups who have been traditionally marginalized. However, as Cornelius et al. (2000) argue, diversity management should be coupled with good equal opportunities policies to be effective and meaningful. This means that valuing the differences of individuals within an organization should be further entrenched by establishing policies and practices that can guarantee their participation and promotion in the workplace. Ensuring that the organization has diverse employee profile increases workplace happiness as it creates an inclusive and welcoming environment conducive to recruiting and retaining the best employees (Cornelius et al., 2000). Mousa (2021) also reveals that gender diversity and diversity management are effective predictors of workplace happiness.

Conversely, employees’ lack of diversity beliefs can lead to organizational bias and rigidity (Turi et al., 2022). This can have long-term impacts on organizational performance. When there is a lack of diversity beliefs in the workplace, decisions may not benefit from a variety of perspectives or reflect the diversity of interests that are both necessary for organizational development. Nevertheless, diversity also has its pitfalls. When diversity is not managed correctly, it can lead to unhealthy organizational politics characterized by misunderstandings, suspicion, distrust, and low morale among employees (Wrench, 2005; Mujtaba, 2007; Turi et al., 2022). Thus, diversity can result in increased or reduced workplace happiness, impacting overall organizational performance. Turi et al. (2022) further note that “a moderate level of gender diversity boosts the competitive edge, whereas greater levels of gender diversity reduce organizational performance” (p. 3). This goes to show that instituting gender diversity requires a balancing act to ensure that differences among employees are appropriately managed to improve both workplace happiness and organizational performance. The study by Blouch and Azeem (2019) conducted in the health sector in Pakistan also reveals that male and female employees are similar in their perceptions of retaining diversity. Given this, it is noteworthy to explore the validity of the following hypothesis in the Bangladeshi context.

H4: Gender diversity mediates the relationship between diversity beliefs and workplace happiness of employees.

### 2.6 Workplace happiness, job satisfaction, engagement and organizational commitment

Happiness at work is a favorable mental state that employees may have while they are at their place of employment (Ryan and Deci, 2001; Fisher, 2010; Field and Buitendach, 2011). Happiness at work significantly impacts a number of organizational characteristics, such as affective organizational commitment (AOC), job satisfaction (JS) and work engagement (WE). These factors markedly impact overall output, staff retention, and the success of the business (Alt, 2021; Algarni and Alemeri, 2023). Alt (2021) argues that the greater the level of employee satisfaction, the higher the likelihood of achieving success for your organization.

**Figure 1:**
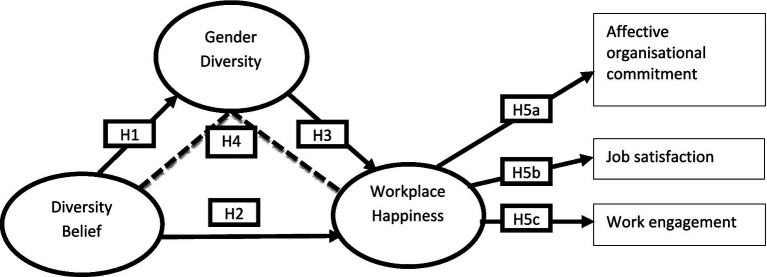
Conceptual model of the study.

Increased employee satisfaction is positively correlated with a heightened sense of loyalty towards the firm. The establishment of an emotional bond between individuals and their organization results in increased levels of *affective organizational commitment (AOC)*, as happy employees are more likely to synchronize their personal objectives with those of the firm (Field and Buitendach, 2011; Wesarat et al., 2014). An upbeat workplace creates an atmosphere where workers are appreciated and treated with dignity. When workers are enthusiastic about what they do for a living, their *job satisfaction (JS)* levels show. Furthermore, happier workplaces frequently result in improved interactions between staff members, management, and co-workers (Fisher, 2010; Roy and Konwar, 2020; Algarni and Alemeri, 2023). The degree to which workers are enthusiastic about their jobs, devoted to their companies, and willing to go above and beyond in their work is known as *work engagement (WE)*. Happy workers are more likely to be engaged, demonstrating zeal and commitment (Fisher, 2010; Field and Buitendach, 2011). Better performance and increased productivity are frequently the outcomes of this high level of engagement. Happy, engaged workers are more inclined to offer fresh perspectives and solutions, which advance the company (Rashmi and Singh, 2020; Algarni and Alemeri, 2023). Given the literature discussed above, we construct the following hypotheses (H5a, H5b, and H5c) (Figure 1).

H5a: Workplace happiness significantly affects organizational commitment.

H5b: Workplace happiness significantly affects job satisfaction.

H5c: Workplace happiness significantly affects work engagement.

## 3 Materials and methods

### 3.1 Sample

The primary goal of the current study is to investigate the experiences of full-time Bangladeshi workers regarding diversity management, as well as how gender diversity influences their workplace happiness related to affective organizational commitment, job satisfaction, and work engagement. To the best of the authors’ knowledge, no prior research has been done on the association between gender diversity, perceptions of diversity management, workplace happiness, work engagement, and job satisfaction—especially in the Bangladeshi work environment. For constructing our sample, we gathered data from public and private sector industries in Dhaka, the capital city of Bangladesh. The sample consists of 320 full-time employees who have worked for at least 2 years in their current workplace, independent of the type of organization, gender identity, religion, civil status or economic level to ensure variations among samples. Exclusion criteria included employees with less than 2 years of experience at their current workplace, a minimum age of 20 and part-time and/or casual employment.

### 3.2 Data collection

The current study draws on primary data using a survey among full-time employees in Dhaka, Bangladesh. Following the principle of simple random sampling, the authors sent out 500 structured questionnaires to potential participants by official email and social media platforms such as Messenger and WhatsApp. After closely monitoring the data collection process, which took place for around 3 months (April 2023 to June 2023), we were able to obtain 330 (66%) completed questionnaires from the participants. However, finally, 320 questionnaires (64%) were deemed suitable for analysis, as 10 questionnaires were found to be defective for diverse reasons.

### 3.3 Measures

The current quantitative study’s conceptual framework was derived from earlier independent research on gender, diversity beliefs, workplace happiness, work engagement, affective organizational commitment, and job satisfaction. For diversity beliefs, we used 7 items of workplace diversity management (see Mor Barak et al., 1998; Ely and Thomas, 2001) after updating to fit the Bangladeshi organizational setting. For gender diversity, we adopted 5 items to elaborate on the effective management of gender diversity, which entails positive organizational outcomes such as enhancing employee commitment, work engagement, etc. (see Larkin et al., 2012; Perrault, 2015; Mousa et al., 2020; Mousa, 2021). We took into consideration the research conducted by Fisher (2010), who explains the three components that make up workplace happiness: work engagement (8 items), job satisfaction (6 items), and affective organization commitment (7 items). All of the items under each construct were scored using the five-point Likert-type scale where 5 indicate strong agreement and 1 indicates strong disagreement of the respondents.

The study used descriptive analysis for the demographic information. It used the Structural Equation Modeling (SEM) technique to analyze and interpret the path relationships based on the hypotheses of the study. The strengths of the data are evaluated using several validity and reliability statistics such as discriminant and convergent validity, composite reliability, Cronbach’s alpha, etc. Some of the model fit indices such as normed fit index (NFI), goodness of fit index (GFI), comparative fit index (CFI), standardized root mean square residuals (SRMR) etc. were also used to assess the fitness of the model.

## 4 Results

### 4.1 Participants’ demographic profiles

Table 1 shows the demographic details of the respondents. Among 320 respondents, 55% were male, and 45% were female. Most of the respondents had work experience of 1–5 years (73%) for their organization and held a bachelor’s degree or higher. This indicates that the participants were well-educated and possessed sufficient skills and knowledge to answer all the survey questions proficiently. The respondents had different organizational backgrounds.

**Table 1:** Demographic profile of the respondents.

**Particulars**	**Frequency**	**Percentage**	**Particulars**	**Frequency**	**Percentage**
**Gender**			**Education**		
Male	176	55	SSC	4	1.3
Female	144	45	HSC	13	4.1
**Total**	320	100.0	Bachelor’s	195	60.9
**Civil Status**			Master’s	107	33.4
Single	145	45.31	Ph. D	1	0.3
Married	158	49.37	**Total**	320	100.0
Divorced/separated	12	3.75	**Type of organisation**		
Widowed	5	1.56	Educational institution	73	22.8
**Total**	320	100.0	Bank and financial institution	50	15.6
**Work Experience**			MNC	12	3.8
2–6 years	234	73.1	NGO/INGO	8	2.5
7–11	37	11.6	Manufacturing	53	16.6
12–16	17	5.3	Research/Consultancy firm	19	5.9
More than 16 years	32	10.0	Service industry	55	17.2
**Total**	320	100.0	Others	50	15.6
			**Total**	320	100.0

### 4.2 Instrument reliability and validity

#### 4.2.1 Reliability

The reliability of the data is measured using Cronbach’s alpha, rho_a and Composite reliability (CR). Theoretically, the values of Cronbach’s alpha, rho_a, and Composite reliability (CR) are said to be satisfactory when these are.70 or above. Table 2 shows that Cronbach’s alpha, rho_alpha composite reliability (CR) values range from 0.773 to 0.946, indicating highly consistent data and a high level of reliability (Chin, 1998; Hair, 2006; Hair et al., 2010, 2022). Furthermore, the values of the goodness of fit indices fell within the acceptable range (Bentler and Bonett, 1980; Hu and Bentler, 1998; Tabachnick and Fidell, 2007; West et al., 2012).

**Table 2:** Construct’s reliability and validity.

Construct	No. of items	Factor loadings	Alpha	rho_A	CR	AVE
AOC	7	0.738–0.854	0.879	0.886	0.907	0.584
DB	6	0.699–0.769	0.797	0.796	0.855	0.596
GD	5	0.742–0.874	0.773	0.824	0.887	0.567
JS	6	0.725–0.868	0.856	0.867	0.894	0.585
WE	8	0.706–0.860	0.872	0.881	0.902	0.569
WH	Higher-order construct		0.939	0.943	0.946	0.557

Model fit indices: Chi square = 208.056, df = 105, Chi square/df = 1.98, GFI = 0.929, NFI = 0.934, TLI = 0.956, CFI = 0.966, SRMR = 0.04, and RMSEA = 0.05. AOC, Affective organizational commitment; DB, diversity beliefs; GD, gender diversity; JS, job satisfaction; WE, work engagement; WH, workplace happiness.

#### 4.2.2 Validity

Validity assessment of the data covered the convergent validity and discriminant validity.

##### 4.2.2.1 Convergent validity

The Convergent validity was assessed by considering the average variance extracted (AVE) and the values of the item loadings. The analysis revealed that, all the values of AVE of the constructs fall between 0.557–0.596 (see Table 2). The AVE values are well above 0.5 and sufficiently met the criteria of convergent validity (Hair, 2006; Hair et al., 2010, 2022). The above Table 2 also shows that all the values of the item loadings are above 0.7 and thus the criterion of the convergent validity was met (Hair et al., 2022).

##### 4.2.2.2 Discriminant validity

Discriminant validity of the constructs was checked using Fornell-Larcker Criterion, cross loading values and the Heterotrait-Monotrait (HTMT) ratio. In case of the FL test, the discriminant validity confirms correlation among constructs if the values do not exceed 0.85 (see Table 3: Fornell-Larcker Criterion) and the square root of AVEs is greater than the correlation of other constructs (Bagozzi et al., 1991; Henseler et al., 2009). Table 3 represents that all values are less than 0.85, and their square root of AVEs was greater than their constructs’ off-diagonal values. These details satisfy the discriminant validity requirements. HTMT ratio refers to the average of the correlations of indicators between different constructs relative to the average of the correlations of indicators within the same construct. It measures the discriminant validity between the construct of the instrument. While conservative cut-off values are 0.9, it advocated a more stringent ratio of 0.85 as it offers the best criterion compared to all other methods of assessing discriminant validity (Henseler et al., 2015). Thus, any inter-construct ratio greater than 0.85 would be considered as having poor discriminant validity. The HTMT ratios obtained in this study, as shown in Table 3, indicate no discriminant validity problems between the constructs. For cross loading, the correlation of a construct with its corresponding items must be higher than that with the items of the other constructs (Henseler et al., 2009). The analysis also fulfilled the requirement of it and hence the data represented the sufficient discriminant validity.

**Table 3:** Fornell-Larcker Criterion (FL-test) and HTMT ratio.

**Fornell-Larcker Criterion (FL-test)**						
Constructs	AOC	DB	GD	JS	WE	WH
AOC	0.765					
DB	0.442	0.704				
GD	0.043	0.055	0.787			
JS	0.731	0.489	0.033	0.765		
WE	0.698	0.479	0.038	0.698	0.754	
WH	0.409	0.521	0.046	0.589	0.592	0.776
**HTMT ratio**						
AOC						
DB	0.523					
GD	0.046	0.08				
JS	0.733	0.589	0.045			
WE	0.793	0.568	0.052	0.794		
WH	0.697	0.598	0.055	0.682	0.784	

### 4.3 Hypothesis testing

The path estimation or hypothetical relations was performed to observe the significant relationship in the inner path model. The entire hypothetical path in the framework was examined through the regression coefficient (β) and t values. The values of the beta coefficient indicate the nature of influence of the independent variable on the dependent variable. T values indicate whether the influence is significant or not. T values above 1.96 specify the significance of the influence. The partial least square Bootstrap technique was used to identify the value of β and t to test the proposed hypotheses in the structural model. Table 4 demonstrates the path coefficient and the t value assessment result, where the analysis revealed that all of the hypotheses were supported. The supported hypotheses were significant at the level of 0.005 (Collier, 2020; Hair et al., 2022) and have positive directions (the beta coefficient is positive), i.e., the independent variables positively affect the dependent variable of the proposed model. Furthermore, the high t values of hypotheses H5a, H5b, and H5c indicate a strong association between the variables. This suggests that as employees’ workplace happiness increases, so does their level of organizational commitment, work engagement, and job satisfaction.

**Table 4:** Results of the hypotheses testing.

Hypotheses and paths	*β*	*T* statistics	*P-*values	Supported
H1: DB - > GD	0.455	2.415	0.003	Yes
H2: DB - > WH	0.520	11.221	0.000	Yes
H3: GD - > WH	0.27	2.371	0.000	Yes
H5a: WH - > AOC	0.909	76.08	0.000	Yes
H5b: WH - > JS	0.889	54.37	0.000	Yes
H5c: WH - > WE	0.892	55.16	0.000	Yes

### 4.4 Mediation hypothesis

**Figure 2:**
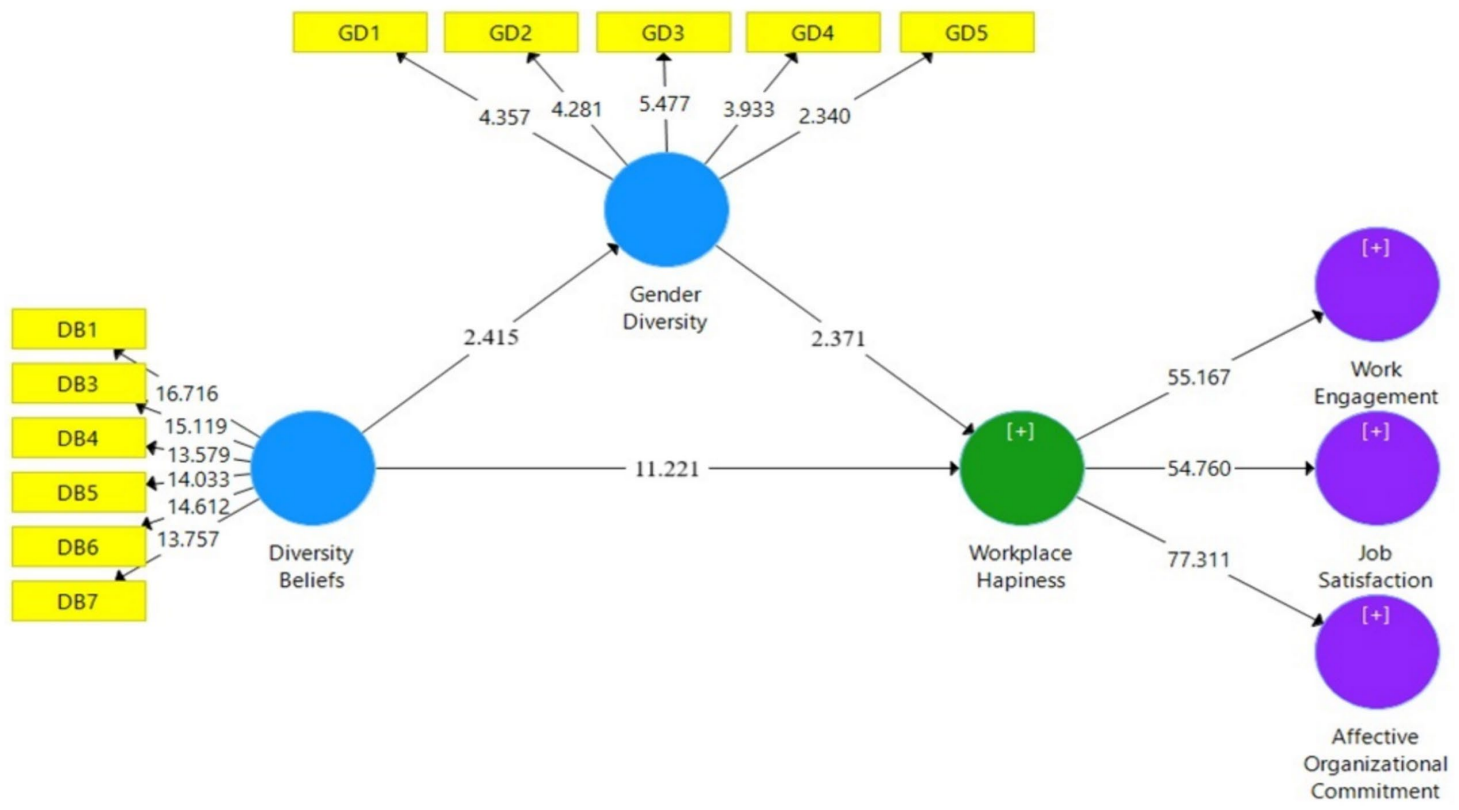
The structural model.

For the mediating analysis, the bootstrapping technique was applied (Zhao et al., 2010). The mediation analysis results are presented in Table 5, where we can see that the effect of the mediating path DB- > GD- > WH is significant at *p* > *0*.05, *t* < 1.96 which confirmed that there is a mediating effect between the variables (Hair, 2006; Hair et al., 2010, 2022; Collier, 2020). And hence, the hypothesis H4 was supported. This means gender diversity has a mediating role between the relationship of the diversity beliefs and the workplace happiness of the employees in the private sector organizations of Bangladesh (Figure 2).

**Table 5:** Mediation analysis.

**Hypothesis and path**	**Direct effect**		**Indirect effect**			**Mediated**
	*β*	*t*	*β*	*t*	*p*	
H4: DB- > GD- > WH	0.520	11.221	0.27	2.587	0.002	Yes

## 5 Discussion

When evaluating employees in the workplace based on factors such as loyalty, satisfaction with work, and level of dedication and engagement, gender and diversity management has become crucial. Much of the earlier research reveals that gender diversity and diversity management undeniably create a better working environment with improved interpersonal relations and understanding. The current study aims to place greater emphasis on the mediating effects of gender on diversity management and workplace happiness in light of the Bangladeshi context. The cross-cultural distinctions between Bangladesh and other nations become very significant and fascinating when these topics are considered in the context of Bangladesh. These variances most certainly play a role in the daily organizational context of the country. Past research on Bangladesh (see Hossain, 2016; Khan et al., 2023) characterized Bangladeshi culture as male-oriented. They found that Bangladeshi managers are human-focused and independent in a male-dominated environment. Our findings demonstrate that Bangladeshi employees’ diversity beliefs positively affect workplace happiness. This study supports the attitudinal theory, in which employees form certain beliefs, emotions and commitments that lead to positive behavior towards their respective organizations.

Furthermore, the outcome is unmistakably consistent with the social exchange theory, which holds that workers’ full potential can only be utilized in organizations if they feel both financial and psychological appreciation from diversity perspectives (Deluga, 1994; Homan, 2019). Therefore, passion, initiative, perseverance, and affiliation for the organization’s improvement are frequently translated into actions that reflect an employee’s sense of engagement, satisfaction with their job, and emotional attachment to their employer. Dobbin and Jung (2011) point out that most empirical research on diversity management and gender has been done in Western nations. Welzel (2014) argues that women in non-Western societies primarily take care of their families and have limited access to the labor market, while women in Western societies who actively participate in various organizational settings are able to hold leadership roles and contribute to research and development. As a result, the importance of diversity management must be recognized, especially for a country like Bangladesh.

The findings further suggest that the workplace happiness of Bangladesh’s employees significantly depends on their diversity beliefs, and the diversity beliefs of the employees help promote gender diversity in the organizations. Findings also indicate that the diverse views of the employees enrich the work environment by enhancing better communication, interpersonal relations, and understanding. As a consequence, this facilitates the workplace happiness of the employees. Similarly, the diversity belief of employees promotes gender diversity in organizations. These findings are aligned with the results of Gao and He (2017) and Bizri (2018). The positive correlation between employees’ diversity beliefs and gender diversity in organizations can be explained by how they outlined how diversity management continuously shapes the managerial actions that secure respect, equality, and engagement of people to achieve organizational and personal goals.

Furthermore, the employees’ direct experiences, beliefs, and perceptions about diversity, jobs, job satisfaction, and other issues facilitate their happiness toward their work. This finding is also consistent with Erdogan et al. (2012), who state that employee satisfaction with leadership, working environment, job description, career growth, and other factors determine the degree of workplace pleasure. Diversity management policies and practices, according to Cho and Mor Barak (2008), Pitts (2009), and Mousa and Puhakka (2019) aim to create an inclusive organizational climate in which gender differences are respected, all employees are treated fairly, and performance is evaluated based on qualifications and performance level. Thus, the diversity beliefs of the employees and proper diversity management guarantee that the female employees have great authority and are not faced with obstacles or worries because of their gender. This is why the diversity belief of employees promotes gender diversity in organizations and increases their job satisfaction and commitment.

Our study finds a significant association between gender diversity and employees’ workplace happiness. The researchers explain that compared to the past several decades, female participation in the workplace of Bangladesh has increased significantly, which is now 38.6% (Zaman, 2023). So, the belief in diversity positively influences the inclusion of gender diversity in Bangladesh’s organizations. Consequently, gender diversity in organizations creates a sound environment that facilitates employees’ workplace happiness. Since workplace happiness factors vary between the perceptions of male and female employees of the private sector organizations of Bangladesh, the influence of gender diversity on workplace happiness is found to be significant; hence, it also mediates the relationship between diversity belief and workplace happiness of the employees. In favor of the finding that the diversity belief of employees positively affects their workplace happiness, the researchers conclude that when employees are aware of diversity-related issues and believe in embracing diversity in organizations, their level of commitment, engagement, and job satisfaction increases. Thus, the belief in diversity has a significant favorable influence on employees’ workplace happiness.

## 6 Theoretical and practical implications

The findings of this study strengthen the relevance of the social exchange theory in analyzing contemporary organizational behavior in the context of diversity management. The findings support the notion that organizations and employees mutually benefit from instituting diversity practices that recognize and value women’s contribution to the workforce. The study’s focus on Bangladesh further proves that the attitudinal theory remains a useful lens in understanding gender dynamics in a male-dominated setting. The study also offers key practical insights on how to ensure effective diversity management. By highlighting the effect of gender diversity on diversity beliefs and workplace happiness, the study calls attention to the need to improve women’s employment access to both public and private organizations, especially in Bangladesh. The study implies that leaders and managers should be more receptive to gender diversity, given the finding that employees who are aware of and believe in diversity management have higher levels of satisfaction, organizational commitment and engagement. Organizations that aim to retain workers should, therefore, develop and promote leaders who embrace gender diversity as a critical goal.

## 7 Limitations and future research directions

Along with its various theoretical and empirical contributions, the current study also has some limitations. The number of participants is limited to only 320 all from Dhaka, though Dhaka is the prime location for workers being the country’s capital city. As a result, our generation may produce less effective assumptions. Future research should expand the number of respondents in the nationwide study and mix different age groups with a specified threshold number for each group. Our data was gathered from a wide variety of private and public organizations. While there are some advantages to these variations in organization selection, data collected in more specific organizational settings can facilitate a more in-depth investigation for future research. The quantitative research we conducted had a moderate response rate, which may restrict the wider generalizations of the results (Sorooshian, 2013). To gain a deeper understanding and more inclusive results, future research might integrate qualitative and quantitative approaches, using both unstructured and structured data (Zahidy et al., 2018). We have demonstrated the mediating effect of gender diversity on diversity beliefs and workplace happiness. However, since Bangladesh is a predominantly Muslim nation, the analysis could have benefited from consideration of the country’s strong religious ties. Future research on the significance of religious variations for diversity management in a traditional nation like Bangladesh can be conducted to address this limitation.

## 8 Conclusion and recommendation

Diversity has recently become one of the most talked-about metrics for measuring success in the workplace. That is why training and raising diversity awareness will result in vigor, improved production, and value creation. To successfully manage diversity in the workplace, one must consider the cultural variations among employees and find ways to leverage and respect these differences to persuade them to work together towards a common objective and do so in a way that gives organizations a competitive advantage. This research has given additional focus on gender diversity as moderating variables. Our results indicate that gender diversity significantly moderates the associations between diversity beliefs and workplace happiness. This means that employees are more likely to be satisfied with their jobs if the workplace reflects the variety of its workforce in terms of gender. Additionally, the current study reveals data supporting the idea that female employees generally have more positive attitudes towards diversity management than their male counterparts. Considering the long history of male dominance in Bangladeshi workplace, it is likely that female employees may find more significant support in challenging masculine control in the workplace, leading to an increase in gender diversity. However, despite the fact that gender diversity in Bangladeshi workplaces has evolved over the years and that more females have entered the labor force, workforce participation and leadership positions continue to be dominated by males. Firms and governments must, therefore, increase their attempts to implement diversity management, including establishing gender-equal policies that appear to be more effective at expanding women’s labor market participation. In nations where the representation of women is comparatively lower, it has been demonstrated that more dynamic public policies are crucial for advancing and addressing the issue of diversity. The Bangladeshi government can draw insights from this study and facilitate a continuing increase in female employment by providing them with legal protection and benefits in the workplace. These may include mandated gender sensitivity training in all organizations and improved provisions for childcare and maternity leave, among others.

## Data availability statement

The original contributions presented in the study are included in the article/supplementary material, further inquiries can be directed to the corresponding author/s.

## Ethics statement

Ethical approval was not required for the study involving human participants in accordance with the local legislation and institutional requirements. Written informed consent for participation was not required for this study in accordance with the national legislation and the institutional requirements.

## Author contributions

SI: Conceptualization, Formal analysis, Funding acquisition, Investigation, Methodology, Project administration, Supervision, Writing – original draft, Writing – review & editing. JA: Conceptualization, Data curation, Formal analysis, Writing – review & editing. MP: Data curation, Resources, Writing – review & editing.
